# The High-Cycle Tensile–Shear Fatigue Properties and Failure Mechanism of Resistance Spot-Welded Advanced High-Strength Steel with a Zn Coating

**DOI:** 10.3390/ma17184463

**Published:** 2024-09-11

**Authors:** Yu Sun, Jiayi Zhou, Rongxun Hu, Hua Pan, Kai Ding, Ming Lei, Yulai Gao

**Affiliations:** 1 State Key Laboratory of Advanced Special Steel, School of Materials Science and Engineering, Shanghai University, Shanghai 200444, China; sunyu00@shu.edu.cn (Y.S.); zhoujy00@shu.edu.cn (J.Z.); rongxunhu@shu.edu.cn (R.H.); dingkaiwsj@shu.edu.cn (K.D.); 2 State Key Laboratory of Development and Application Technology of Automotive Steels, Baosteel Group, Shanghai 201900, China; hpan@baosteel.com; 3 Automobile Steel Research Institute, R&D Center, Baoshan Iron & Steel Co., Ltd., Shanghai 201900, China

**Keywords:** LME cracks, tensile–shear fatigue, crack initiation, stress concentration, resistance spot welding

## Abstract

Advanced high-strength steels (AHSSs) with Zn coatings are commonly joined by the resistance spot welding (RSW) technique. However, Zn coatings could possibly cause the formation of liquid metal embrittlement (LME) cracks during the RSW process. The role of a Zn coating in the tensile–shear fatigue properties of a welding joint has not been systematically explored. In this study, the fatigue properties of tensile–shear RSW joints for bare and Zn-coated advanced high-strength steel (AHSS) specimens were comparatively studied. In particular, more severe LME cracks were triggered by employing a tilted welding electrode because much more stress was caused in the joint. LME cracks had clearly occurred in the Zn-coated steel RSW joints, as observed via optical microscopy. On the contrary, no LME cracks could be found in the RSW joints prepared with the bare steel sheets. The fatigue test results showed that the tensile–shear fatigue properties remained nearly unchanged, regardless of whether bare or Zn-coated steel was used for the RSW joints. Furthermore, Zn mapping adjacent to the crack initiation source was obtained by an electron probe micro-analyzer (EPMA), and it showed no segregation of the Zn element. Thus, the failure of the RSW joints with the Zn coating had not initiated from the LME cracks. It was concluded that the fatigue cracks were initiated by the stress concentration in the notch position between the two bonded steel sheets.

## 1. Introduction

Lightweight yet high-strength steels with the superior incorporation of mechanical properties such as strength and ductility are continually designed in automotive industry applications to meet the demands of safety, energy-efficient vehicle development, and sustainable economic growth, especially for third-generation advanced high-strength steels (AHSSs) [1,2,3]. In particular, the resistance spot welding (RSW) technique is generally employed to join AHSS sheets to manufacture automotive white bodies. The RSW technique, which is a rapid joining technique in high-volume and/or high-rate automation production without filler metals or fluxes, is extensively applied, with approximately 5000~8000 spot welds in one automotive white body [4,5,6]. Therefore, understanding the RSW process and evaluating RSW performance in AHSS joining is significant for the integration of this welding technique into industry.

The RSW process comprises metal surface contact and bonding via the heat obtained from the resistance to an electric current flow, which is an interaction involving mechanical, metallurgical, thermal, and electric phenomena, among others [7]. From a mechanical and electrical perspective, for example, different electrode geometries and angular welding electrode misalignments could affect the electrical contact resistance and stress concentration [8,9,10,11]. Metallurgical and mechanical phenomena related to the varied and complex interface between the nugget, heat-affected zone (HAZ), and base metal (BM) can trigger dramatic fluctuations in material hardening, microstructure, and phase transformation [12]. Further, RSW process parameters have been focused and are combined with the mechanical properties of weld joints. The diameter and quality of the nugget are characteristics that are influenced by important process parameters, e.g., the welding current and welding time [13,14]. In addition, liquid metal embrittlement (LME) can occur during the RSW process in some corrosion-resistant Zn-coated steels. Certain conditions regarding temperature and stress can result in the Zn penetrating the grain boundaries, and this can lead to the formation of LME cracks [15]. Unfortunately, an efficient approach to suppress LME cracks has, until now, been controversial. Furthermore, in-depth studies are required to investigate the existence of LME cracks and their influence on the mechanical behavior of RSW joints. In particular, high-cycle fatigue property measurement is necessary to evaluate the performance of joints with LME cracks.

As for the failure analysis of the RSW joint, overload failure and fatigue failure are two important mechanisms that occur, according to vehicle crashworthiness standards, under different driving conditions. Also, the failure mode is a criterion used to evaluate the quality of RSW joints. There are three failure modes: interface failure (IF), partial interface failure (PIF), and pull-out failure (PF) [16]. The desirable mode of pull-out failure occurs with higher plastic deformation and energy absorption than the other two failure modes. The variation in the failure mode significantly depends on the nugget size. Some researchers have reported that the pull-out failure mode is more common for large-size nuggets, exhibiting a higher peak load and energy absorption, whereas the interface failure mode is more typical of smaller nuggets [17,18,19]. In addition to the factor of nugget size, the effect of LME cracks on RSW joint strength has also been widely investigated. Zhang et al. reported that the strength of the RSW joint for TRIP (transformation-induced plasticity) steel with LME cracking was correlated with the nugget/BM (base metal) hardness ratio [20]. DiGiovanni et al. indicated that the LME crack size and location (i.e., the outer surface, inner surface, and zone with the critical load) could affect the RSW joint strength to a certain degree [21,22]. LME cracks longer than 400 μm and located in the outer surface edge caused a loss of peak load and energy absorption in tensile–shear testing. Wintjes et al. proposed an LME crack index characterizing the crack size and location, which fit a nearly linear curve with the tensile–shear strength loss [23]. Conversely, Benlatreche et al. [24] and Kwon et al. [25] stated that LME cracks did not affect the tensile–shear strength. Regarding the Zn-coated RSW joint of TRIP steel, fatigue testing was conducted using a tensile–shear specimen, and the results showed that fatigue life was hardly influenced by the LME crack yet was proportionate to the nugget size [26]. Based on fracture observations with the fatigue propagation characteristics of beach marks, Gaul et al. noticed that the area of the LME crack in the HAZ was much smaller than that of the fatigue crack, implying that there was no effect of LME cracking on the fatigue properties [27]. Kwon et al. deemed that a type-C LME crack (formed as an inner crack between steel sheets) in the TRIP1180 steel RSW joint could produce a larger effect on the cross-tensile fatigue strength than on the tensile–shear fatigue strength [25]. Jang et al. noted that the type-B crack (formed at the surface edge of the indentation zone) had a negligible effect on fatigue life, and the simulation results revealed that the type-B crack could hardly change the local stress concentration [28].

Undoubtedly, the above-mentioned studies have already exhibited the correlation between fatigue properties and LME cracks in various steel systems. However, little information is available on the initiation of fatigue cracks and their relation to LME cracks. For high-cycle fatigue properties, the nature and position of the initiation of fatigue cracks are crucial factors in explaining the fatigue properties of the RSW joint. Therefore, the present study compared the tensile–shear fatigue properties of the RSW joints of bare AHSS and those with Zn coatings. The type of fatigue crack initiation was determined, and its correlation with LME cracks was investigated by the comparative analysis of the fatigue fracture and cross-sectional morphology. In addition, the failure of the tensile–shear specimen was employed to simulate the stress distribution and elucidate the failure mechanism and its effects on fatigue performance.

## 2. Materials and Experiments

**Figure 1:**
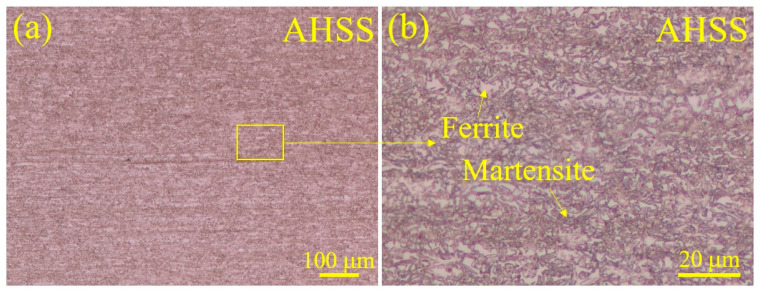
Micrographs of the as-prepared AHSS sheet: (**a**) an image of the metallographic structure and (**b**) a magnified image of the metallographic structure.

**Figure 2:**
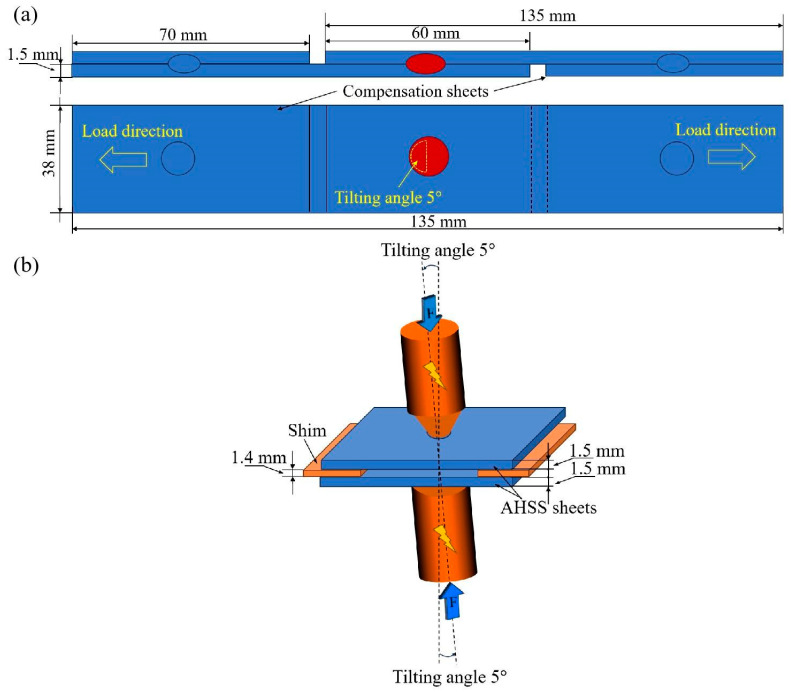
A schematic of the RSW joint of the tensile–shear specimen: (**a**) the dimensions of the RSW joint of tensile–shear specimen and (**b**) the schematic details of the resistance spot welding process in the region near the spot weld.

The chemical composition and mechanical properties of the AHSS sheet are listed in Table 1 and Table 2, respectively. The microstructure of AHSS is shown in Figure 1. The base metal consisted of martensite (dark brown) and ferrite (white). AHSS sheets with a thickness of 1.5 mm were employed as the base metals. The Zn-coated AHSS sheets were galvanized according to standard industrial processing. For the tensile–shear RSW joint specimen, both AHSS sheets were cut with a length of 135 mm, width of 38 mm, and overlap length of 60 mm (Figure 2a). Subsequently, the sheets were welded accurately so that the rolling direction of the steel sheets was parallel to the force and the force load was balanced on both sides during the tensile–shear test. Additionally, compensation sheets with the same thickness of 1.5 mm and length of 70 mm were welded to reduce possible sheet bending and nugget rotation during the tensile–shear test.

The experimental configuration was set according to the corresponding standard (ISO 14324:2003) [29]. The fatigue tensile–shear test was carried out at room temperature by a fatigue testing machine (MTS 370) equipped with a charge cell of +/− 15 kN. Tests were performed with a frequency of 20 Hz by using a sinusoidal waveform. The applied load ratio during the test cycles was set at *R* = *F*_min_/*F*_max_ = 0.1. The loading values were designed to ensure that the fatigue life ranged from 10^3^ to 10^7^ based on the load reduction method. The maximum loading level was set as the yield load compared to the quasi-static tensile–shear RSW joints. The fatigue cycles were counted until the displacement of the testing machine was beyond 2 mm.

**Figure 3:**
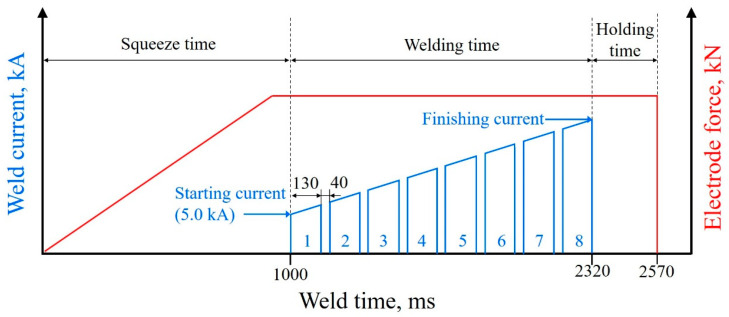
A schematic of the applied resistance spot welding procedure with the electrode force and current profile for 8 progressive sloping impulses.

The joint was welded using the resistance spot welding technique. The welding process parameters were the same except for the welding current. In addition, the misalignment of the welding electrode and an expanded sheet interval were implemented with the aim of increasing the LME crack formation tendency [10]. Thus, a welding electrode with a tilt angle of 5° in the direction opposite to the load side and a sheet interval of 1.5 mm were designed to focus on the effect of LME cracks, as shown in Figure 2b. The welding current mode was 8 progressive sloping impulses with a starting welding current of 5.0 kA and different finishing welding currents. The used RSW procedure is displayed in Figure 3. There was similar welding expulsion between Zn-coated steel and bare steel, while the finishing welding current was 11.6 kA for Zn-coated steel and 10.7 kA for bare steel, generally defined as above the expulsion . A stress simulation was conducted via SORPAS 3D (v6.0) software to show the stress distribution of the welded joint during the tensile–shear test. Macroscopic observations and failure analysis were conducted by using an automated digital microscope (Smart Zoom 5, Zeiss, Oberkochen, Germany). To further analyze the failure characteristics, a metallographic specimen was made by recovering the whole failed joint and then cutting adjacent to the fatigue initiation site. The microstructural details of the magnified region were elucidated by using optical microscopy (OM, Axioscope 5, Zeiss, Oberkochen, Germany) and scanning electron microscopy (SEM, GeminiSEM 300, Zeiss, Oberkochen, Germany). The element distribution details were analyzed by applying an electron probe micro-analyzer (EPMA, JXA-iHP200F, JEOL, Tokyo, Japan).

## 3. Results and Discussion

### 3.1. Quasi-Static Test of RSW Joints

**Figure 4:**
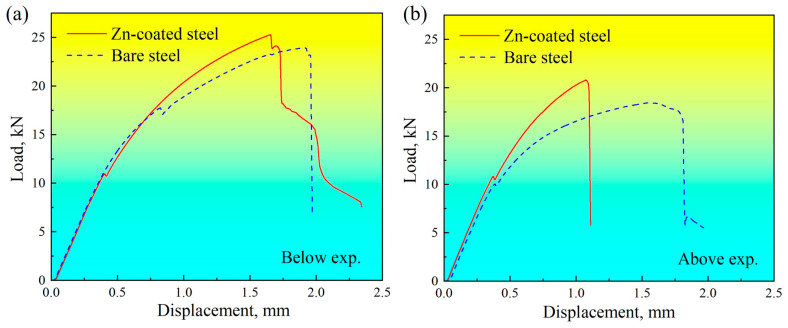
Load–displacement curves obtained by quasi-static tensile–shear loading tests for Zn-coated and bare steel RSW joints: (**a**) below expulsion and (**b**) above expulsion.

Figure 4 shows the load–displacement curves obtained by the quasi-static tensile–shear loading tests for Zn-coated and bare steel RSW joints welded below and above the expulsion. The peak loads of Zn-coated and bare steel joints welded below the expulsion were 25.3 kN and 23.9 kN, respectively. The mechanical properties of Zn-coated and bare steel joints welded below the expulsion were similar. The peak loads of Zn-coated and bare steel joints welded above the expulsion were 22.5 kN and 18.5 kN, respectively. It seemed that joints welded above the expulsion with a severe nugget change could markedly influence the mechanical properties of Zn-coated and bare specimens.

### 3.2. Tensile–Shear Fatigue Test of RSW Joints

**Figure 5:**
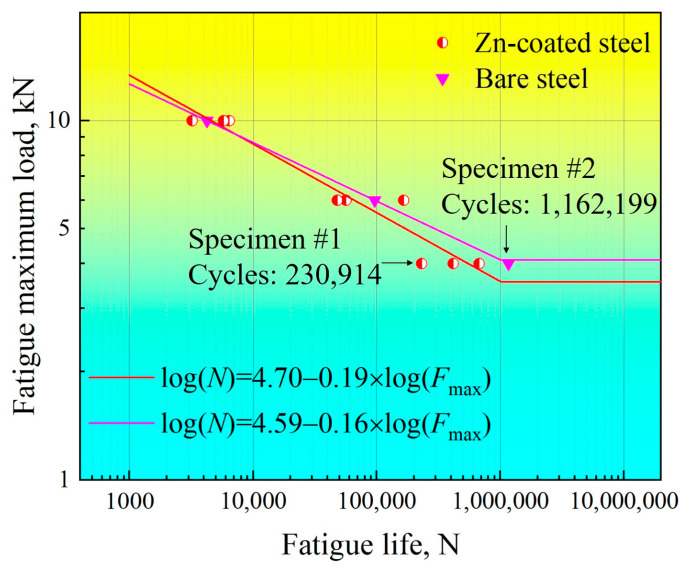
*F-N* curves of the tensile–shear specimens for the RSW joints of Zn-coated and bare steels.

Fatigue tests were performed on two groups of tensile–shear specimens of RSW joints in order to evaluate the effect of LME on fatigue properties after galvanizing, as presented in Figure 5. To investigate the fatigue properties more quantitatively, the fatigue test data were fitted to curves in logarithmic coordinates according to the Basquin Equation (1) [30]. Equation (2) is the fitted equation for Zn-coated specimens, and (3) is for bare specimens, expressed as follows:

lg*N* = *a* + *c* × lg*F* (1)

lg*N* = 4.70 − 0.19 × lg*F* (2)

lg*N* = 4.59 − 0.16 × lg*F* (3)

where *N* is the fatigue cycles, *F* (with the unit kN) is the maximum force load, and *c* is the slope of the fitted curve. The fatigue limits (*F*_L_) were substituted by the maximum fatigue force, providing 10^6^ cycles for the fitted curves. The fitted results indicated that the fatigue limit of the Zn-coated steel RSW joint was *F*_L_~3.58 kN, and that of the bare steel RSW joint was *F*_L_~4.10 kN. The absolute value of the Zn-coated steel RSW joint was |*c*|~0.19, and that of the bare steel RSW joint was |*c*|~0.16. It seemed that galvanizing did not significantly influence the fatigue properties because the fitted curves of Zn-coated and bare specimens were similar. To shed more light on the correlation between the fatigue properties and characteristics of the Zn-coated steel RSW joints, fatigue initiation was emphasized by careful fatigue observations.

### 3.3. LME Observation on the Spot Surface

**Figure 6:**
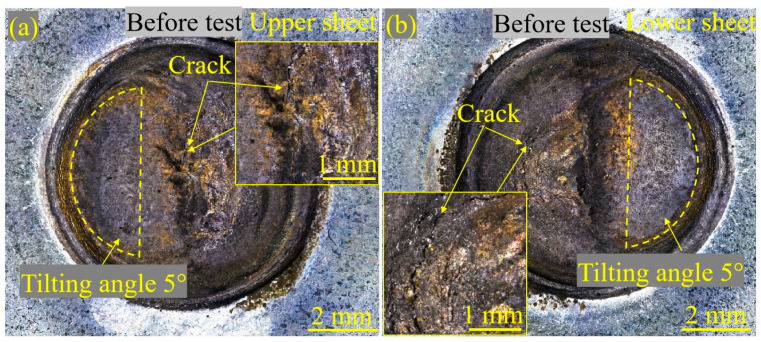
The appearance of the Zn-coated steel RSW joint of tensile–shear specimen #1 before the fatigue test from the views of the two sides’ sheet surfaces: (**a**) upper-sheet image and (**b**) lower-sheet image. The insets in (**a**,**b**) focus on the existence of LME cracks.

Generally, LME cracks appeared on the surface of Zn-coated steel RSW joints welded at and/or above the expulsion. Figure 6 shows the appearance of the Zn-coated steel RSW joint of tensile–shear specimen #1 before the fatigue test from the views of the upper and lower sheet surfaces. It was clear that there was one side on which the electrode had a 5° tilt angle. Clearly, some severe LME cracks can be observed on the surface. The influence of these LME cracks on fatigue crack initiation was further studied, as described below.

### 3.4. Fatigue Crack Initiation Determination

**Figure 7:**
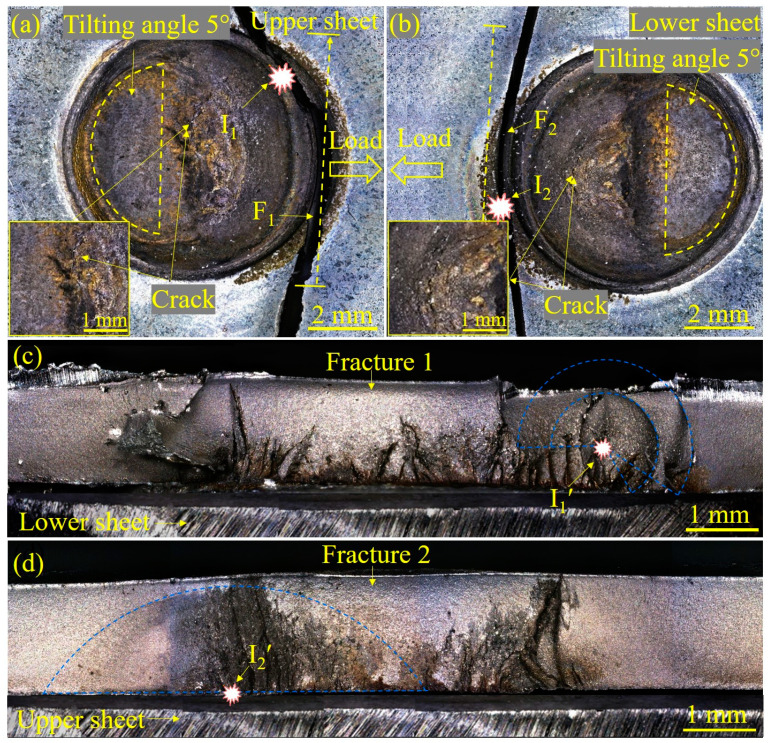
The appearance of the Zn-coated steel RSW joint of tensile–shear specimen #1 after a fatigue test from the views of the sheet surfaces and fracture morphology: (**a**) upper-sheet image, (**b**) lower-sheet image, (**c**) fracture of (**a**), and (**d**) fracture of (**b**). I is the abbreviation for the initiation source determined from the surface, and I′ is that determined from the fatigue fracture. The insets in (**a**,**b**) focus on the existence of LME cracks.

**Figure 8:**
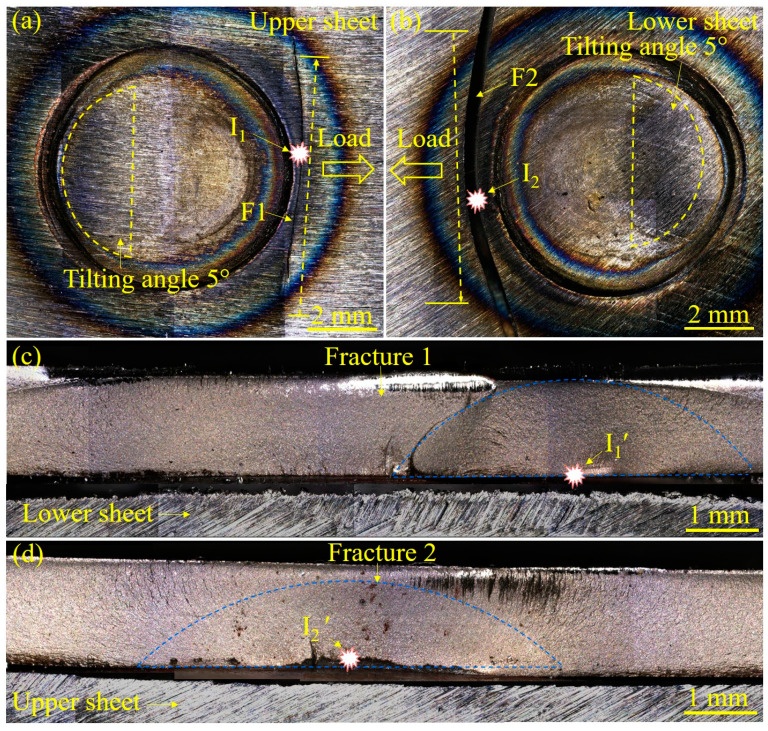
The appearance of the bare steel RSW joint of tensile–shear specimen #2 after a fatigue test from the views of the sheet surfaces and fracture morphology: (**a**) upper-sheet image, (**b**) lower-sheet image, (**c**) fracture of (**a**), and (**d**) fracture of (**b**).

To shed light on the effect of LME cracks on fatigue crack initiation, the appearance of fractured Zn-coated and bare steel RSW joints after the fatigue test is displayed in Figure 7 and Figure 8, respectively. The cracks always occurred on the side of the sheet where the force load was applied. Accordingly, the cracks did not start from the side opposite to the spot because the stress there was small. For Zn-coated steel specimen observations (Figure 7a), the fatigue crack initiated away from the center of the nugget and propagated in a circum-line direction of the nugget. Then, the joint failed in a nearly straight-line direction perpendicular to the load direction to the base metal. The typical characteristics of fatigue crack initiation and propagation can also be observed in Figure 7b. Some studies have pointed out that the majority of fatigue life is spent in the crack initiation stage under high-cycle loading [31]. In this study, the determination of the initiation source and the location of fatigue cracks was the focus. Generally, a semi-elliptic arc shape could be observed for the through-the-thickness fatigue crack fracture morphology. With this strategy, the center of this kind of circular region could be determined as the fatigue crack initiation source (I′). In Figure 7a,b, the fatigue crack initiation sources (I_1_ and I_2_) of the fractured Zn-coated steel RSW joint (#1) are observed from the views of the sheet surfaces. In Figure 7c,d, the fatigue crack initiation sources (I_1_′ and I_2_′) of the fractured Zn-coated steel RSW joint (#1) are observed from the views of the fractures. These comparative observations revealed that all fatigue cracks in the fractured Zn-coated steel RSW joint initiated from the lower part of the fracture, located between the two sheets. From the views of the sheet surface (Figure 8a,b), the fatigue cracks in the fractured bare steel RSW joint (#2) initiated from I_1_ and I_2_, which were much farther away from the center of the nugget than those of joint #1. The fatigue cracks propagated, and ultimately, the joint failed in a straight-line direction nearly perpendicular to the load direction to the base metal. From the views of the fractures (Figure 8c,d), the fatigue crack initiation sources (I_1_′ and I_2_′) of the fractured bare steel RSW joint (#2) were also located between the two sheets.

### 3.5. Analysis of the Failure Mechanism

**Figure 9:**
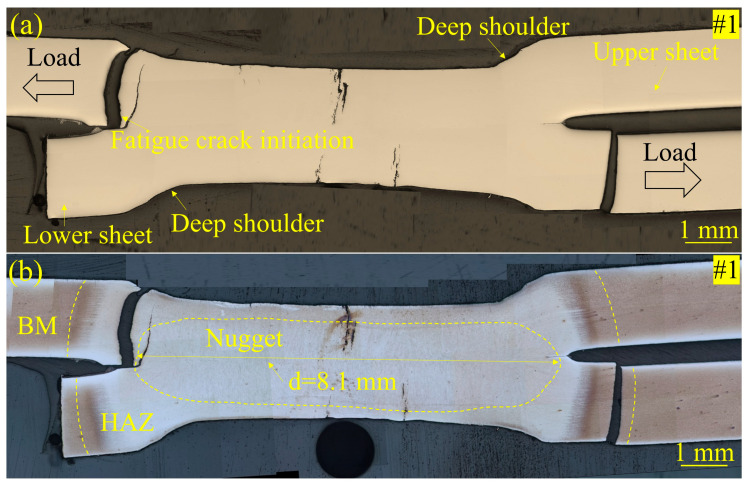
Micrographs of the Zn-coated steel RSW joint of tensile–shear specimen #1 after a fatigue test: (**a**) without chemical etching and (**b**) with chemical etching.

**Figure 10:**
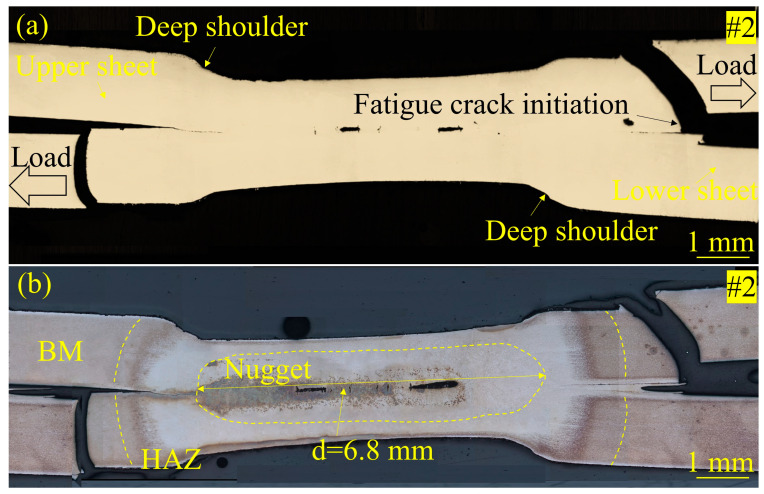
Micrographs of the bare steel RSW joint of tensile–shear specimen #2 after a fatigue test: (**a**) without chemical etching and (**b**) with chemical etching.

Furthermore, a failure analysis of the fractured specimens was performed by OM, with microstructural details being chemically etched. As shown in Figure 9a and Figure 10a, the fatigue cracks normally initiated at the notch site at the interface between the two sheets. For the Zn-coated specimen (#1) shown in Figure 9b, the fatigue fracture first occurred in the HAZ under a high tensile–shear force load. The crack propagated through the HAZ in a plane nearly perpendicular to the steel sheet surface. This kind of fracture is defined as a pull-out failure mode, depending on the mechanical resistance of both the HAZ and nugget. The severe LME cracks in the Zn-coated specimen can be clearly observed in the section plane. However, these LME cracks did not act as fatigue crack initiation sites or their propagation paths. The nugget button was pulled out when the crack in each sheet propagated symmetrically on both sides of the two joined sheets. The nugget size of specimen #1 was measured to be 8.1 mm. For the bare specimen (#2), no LME cracks can be traced through the section plane observation in Figure 10a. As shown in Figure 10b, the crack initiated from the BM and propagated through it to the steel sheet surface. The nugget size of specimen #2 was measured to be 6.8 mm. Similar to the Zn-coated specimen (#1), specimen #2 also exhibited a pull-out failure mode. That is to say, these two kinds of specimens revealed a similar fatigue fracture mode, whether the Zn coating existed or not.

### 3.6. Analysis of the Fatigue Fracture

**Figure 11:**
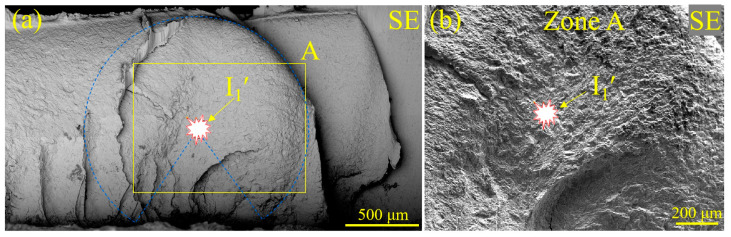
SEM images of the fracture morphology of tensile–shear specimen #1 for the Zn-coated steel RSW joint: (**a**) the second electron image and (**b**) a magnified image of the crack initiation source.

**Figure 12:**
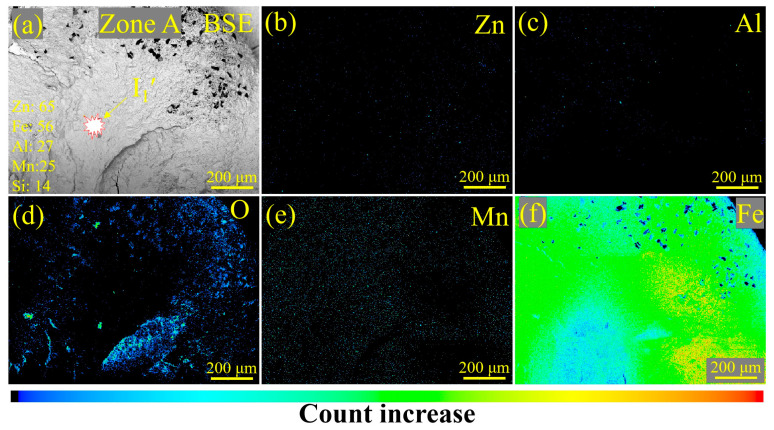
EPMA images of the fracture morphology and element distribution of tensile–shear specimen #1 for the Zn-coated steel RSW joint: (**a**) a back-scattering electron image of the crack initiation source, (**b**–**f**) the distribution of elements Zn, Al, O, Mn, and Fe, respectively. The legend in (**a**) lists the atomic weights of the main elements involved in the steel and the Zn coating.

To further investigate the influence of LME on the fatigue fracture of RSW joints prepared with steel sheets with or without Zn coating, the characteristics of the fatigue fracture were analyzed via SEM and an EPMA. In Figure 11a, fatigue crack initiation (I_1_′) was determined according to the circular arc shape of the crack front (see Figure 7c). From the magnified observation of the I_1_′ zone in Figure 11b, no obvious defects, e.g., inclusions and voids, can be observed. Then, the crack initiation source of the Zn-coated specimen (#1) was further examined via the EPMA, and the results are shown in Figure 12. No Zn existed, according to the element distribution of Zn, Al, O, Mn, and Fe, in the fatigue fracture around the crack initiation source. Thus, it is reasonable to conclude that the existence of LME cracks was not a crucial factor in initiating the fatigue failure of the Zn-coated RSW joints.

**Figure 13:**
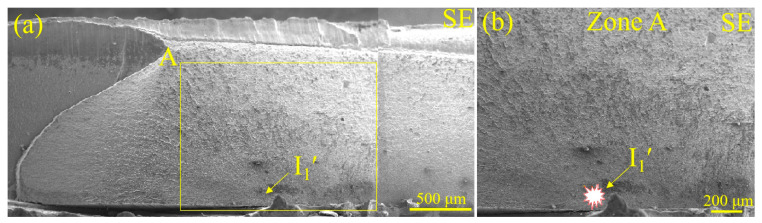
SEM images of the fracture morphology of tensile–shear specimen #2 of the RSW joint for bare steel: (**a**) the second electron image and (**b**) a magnified image of the crack initiation source.

**Figure 14:**
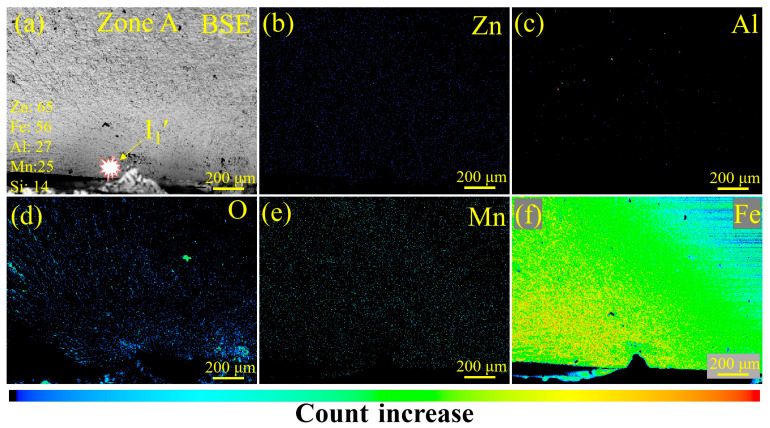
EPMA images of the fracture morphology and element distribution of the bare steel RSW joint of tensile–shear specimen #2: (**a**) a back-scattering electron image of the crack initiation source and (**b**–**f**) the distribution of elements Zn, Mn, O, Al, and Fe, respectively.

To compare the characteristics of crack initiation in the joint without the Zn coating, the initiation source in the bare specimen (#2) was determined and is magnified in Figure 13. Also, no defects could be observed in the zone around the initiation source. The element distribution results presented in Figure 14 further confirm the non-existence of the Zn element.

### 3.7. Stress Simulation for the Tensile–Shear Fatigue Test

**Figure 15:**
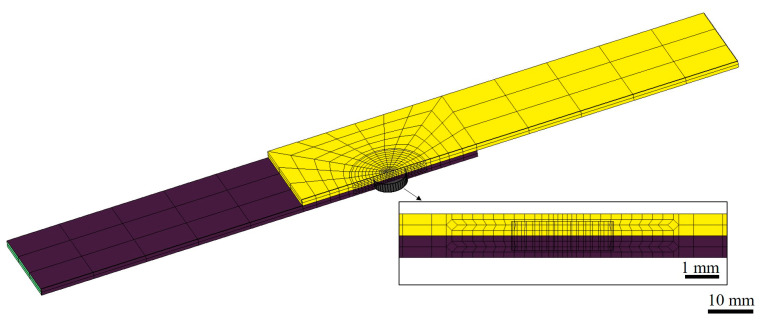
An overview and inset of the simulation model of the tensile–shear specimen for the RSW joint.

**Figure 16:**
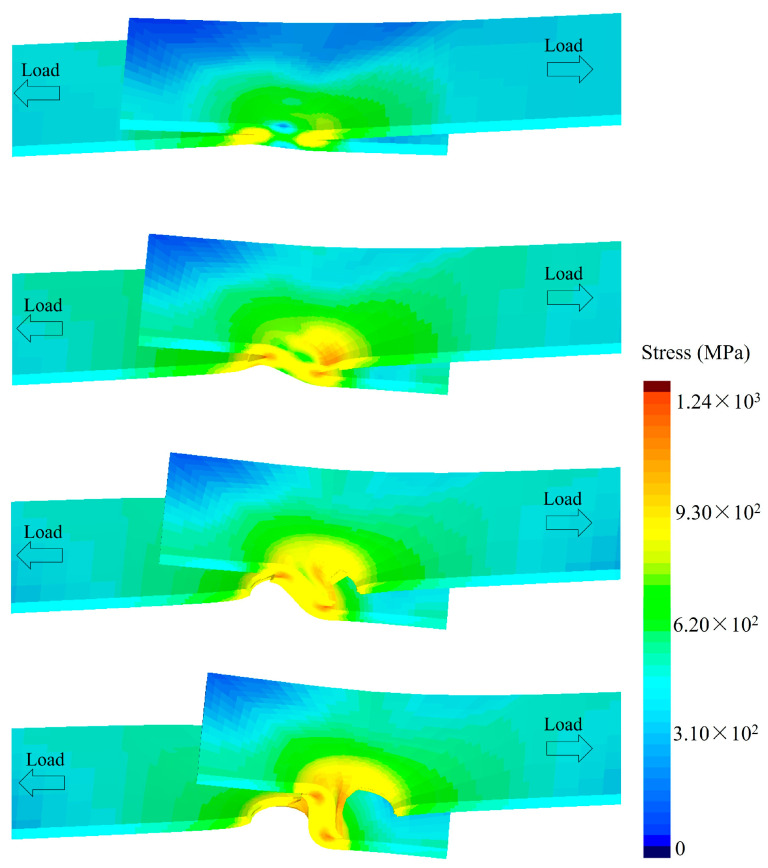
The stress distribution on the tensile–shear specimen for the RSW joint during the tensile–shear fatigue test.

With the help of fracture analysis, it was found that the fatigue crack initiated at the notch in the nugget-surrounding zone and the interface between the two sheets, implying that other factors worked to trigger the fatigue fracture. As a result, the stress concentration around the weld spot during the tensile–shear fatigue test was numerically simulated. To increase the numerical efficiency, half of the typical geometry of the tensile–shear RSW joint was modeled. An overview and inset of the simulation models are shown in Figure 15. The distribution of the von Mises stress near the weld spot is shown in Figure 16. In the direction parallel to the interface, the maximum stress appeared at the position along the perimeter of the weld spot close to the force load. Meanwhile, the stress distribution in the thickness direction needed to be considered to explain the crack initiation that occurred between the two sheets. Clearly, there was severe stress on the interface plane, with the maximum stress occurring at the position of the notch. On the contrary, the stress on the plane near the surface was small. The maximum von Mises stress could be reached due to both the maximum shear stress τ and stress σ arising from the bending moment [32,33]. Thus, crack initiation first occurred between the two sheets at the position of the intersection of the force load centerline and spot perimeter.

## 4. Conclusions

RSW joints of Zn-coated AHSSs with severe LME cracks were prepared by a specially designed welding technique to investigate the influence of the produced LME cracks on the tensile–shear fatigue properties. Joints formed under the same welding conditions were compared for Zn-coated and bare steels. Based on the experimental and simulation results, the following main conclusions were drawn:

(1) The value of the peak load of the Zn-coated joint welded below the expulsion was 25.3 kN, similar to that of the bare joint, which was 23.9 kN. The value of the peak load of the Zn-coated joint welded above the expulsion was 22.5 kN, slightly higher than that of the bare joint, which was 18.5 kN. It seemed that galvanizing did not significantly influence the quasi-static mechanical properties of Zn-coated and bare steel joints below expulsion. However, there were obvious variations in quasi-static mechanical properties for RSW joints above expulsion. 

(2) The slope values of the fitted curved (|*c*|) of Zn-coated and bare steel joints were 0.19 and 0.16, respectively. The fatigue limits (*F*_L_) of Zn-coated and bare steel joints were 3.58 kN and 4.10 kN, respectively. Surface and cross-section observations of the Zn-coated specimen (#1) showed that severe LME cracks appeared in the center of the indentation, which was promoted by increasing the sheet interval and tilting the welding electrode. On the contrary, no LME cracks existed in the bare specimen (#2).

(3) High-cycle fatigue tests revealed that the slope value of the fitted curve (|*c*|) was slightly larger and the fatigue limit (*F*_L_) was slightly lower in the Zn-coated specimen than in the bare one. Nevertheless, it was concluded that no obvious differences existed between the two compared systems, whether LME cracks existed or not. 

(4) The crack initiation source was determined to be a non-defect type by element distribution analysis and fatigue fracture observations, reflecting that the LME cracks were not a crucial factor leading to the fatigue fracture of the Zn-coated RSW joint. 

(5) The numerical simulation results demonstrated that there was an obvious stress concentration at the notch position of the joint, which triggered the initiation of the fatigue cracks. That is to say, the stress concentration during the fatigue test was the crucial factor leading to the fatigue fracture for both the Zn-coated and bare steel joints.

## Figures and Tables

**Table 1:** The chemical composition of the as-prepared AHSS sheet (wt.%).

Elements	C	Mn	Si	P	S	Fe
AHSS	>0.2	>2.2	>0.8	<0.03	<0.01	Bal.

**Table 2:** Mechanical properties of the as-prepared AHSS sheet.

Mechanical Properties	Tensile Strength (MPa)	Yield Strength (MPa)	Elongation (%)
AHSS	1035	724	20.6

## Data Availability

No data were used for the research described in the article.
